# Own and others’ confidence in social information use

**DOI:** 10.1016/j.isci.2026.115968

**Published:** 2026-05-22

**Authors:** Andrea Gradassi, Wouter van den Bos, Lucas Molleman

**Affiliations:** 1 Department of Psychology, University of Amsterdam, Amsterdam 1001 NK, the Netherlands; 2 Institute of Cognitive Sciences and Technologies, National Research Council of Italy, Rome 00196, Italy; 3 Center for Adaptive Rationality, Max Planck Institute for Human Development, Berlin 14195, Germany

**Keywords:** social sciences, psychology

## Abstract

The behavior of friends, colleagues, television hosts, and social media feeds affects what we buy, how we dress, and who we vote for. The confidence with which those opinions are expressed shapes how much weight individuals give them. Yet little is known about how one’s own confidence and others’ confidence jointly determine social information use. We present results of two incentivized decision-making tasks (*N* = 203 and *N* = 213, samples from the U.S.A.) where participants could adjust their initial judgments upon observing judgments of peers and their confidence. Adjustments were most sensitive to the confidence of others when participants’ own confidence was low. Confidence also affected heuristic strategies: confident others prompted participants to compromise and copy social information more often, rather than to stick with their initial estimates. We discuss how following confident others when uncertain can improve decision-making, but also leaves people vulnerable to sources of misinformation.

## Highlights

• Own and others’ confidence jointly shape social information use
• Confident peers are more influential when individuals lack confidence in their own beliefs
• Low self-confidence increases compromise toward and copying of social information
• Following confident others when uncertain may increase vulnerability to misinformation

## Graphical abstract

**Figure:**
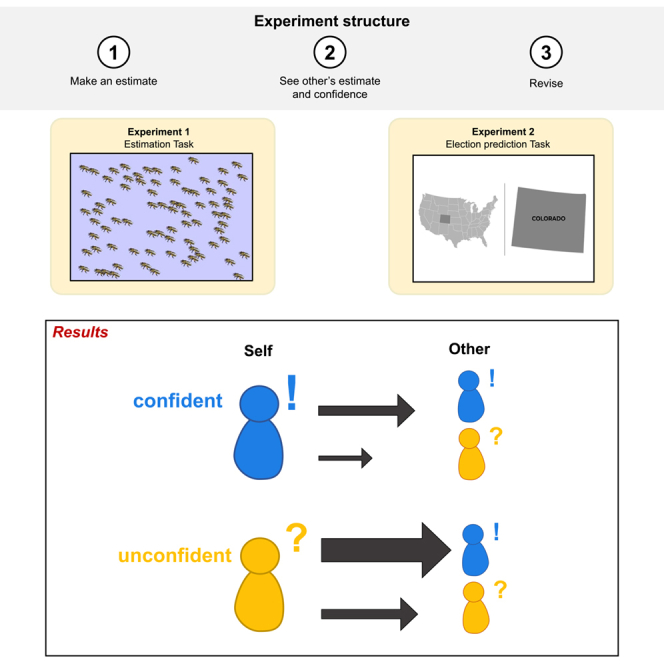

## Introduction

Humans are constantly exposed to the behavior and opinions of others. Such social information can help individuals adjust to new social circumstances and improve the accuracy of their decisions.^1^ Social information is often accompanied by a certain degree of confidence: TV anchors and politicians take assertive postures, consultants are rarely unsure when offering advice, and social media feeds abound in opinionated comments. When the expressed confidence reflects an accurate belief, it facilitates effective decision-making: confident witnesses can assist juries in identifying the right suspects,^2^ and confident doctors can enable medical teams to arrive at correct diagnoses.^3^ However, when high confidence is unsupported by facts, as often is the case in those holding dogmatic or extreme views,^4^ misleading information may readily spread, exacerbating polarization, threatening the adoption of expert medical advice, and potentially swaying election outcomes.^5^^,^^6^^,^^7^ It is therefore crucial to understand when and how individuals use confidence (their own and that of others) to update their beliefs.

Belief updating in social contexts is a balancing problem: “How much weight should I give to my own beliefs relative to those of others?” This question has been studied with judgment tasks in which participants can update their responses after receiving social information.^8^^,^^9^^,^^10^ In such tasks, all else being equal, the optimal strategy is to take the average of one’s own judgment and social information. Observed behavior, however, regularly deviates from this normative expectation. People tend to place greater weight on their own judgment than those of others (i.e., egocentric discounting^11^; foregoing opportunities to improve their judgments. Communicating how confident people are in their judgments, gives additional information about the relative quality of personal and social information and can help solve this balancing problem.

Here, we define confidence as the subjective belief in the accuracy of a judgment.^12^^,^^13^ In the literature, two main mechanisms have been identified for how confidence impacts social information use. According to metacognitive control models, confidence reflects an internal monitoring of one’s accuracy,^14^ which can be done through introspection. Ample empirical evidence indicates that when unconfident, people tend to search more for social information^15^^,^^16^^,^^17^ and rely more heavily on social information in making decisions.^18^^,^^19^^,^^20^^,^^21^ Concurrently, the reliability of social information may be gauged from the confidence expressed by its source, based on non-verbal or verbal expressions (e.g., stating “I’m sure” or using a loud voice). Social dominance and communication theories emphasize how confidence is effective as a cue of competence.^22^^,^^23^ Evidence suggests that this is indeed the case: when a statement is expressed with high confidence, it tends to have a strong impact on the receiver.^23^^,^^24^^,^^25^^,^^26^

In everyday life, however, the confidence of self and others simultaneously shape social information use; for example, people may disregard firm opinions if they clash with their own confidently held beliefs.^27^ The interaction between one’s own and others’ confidence has been explored in perceptual decision-making, where dyads or groups collaborate to detect features of visual stimuli, such as their orientation.^24^^,^^28^^,^^29^ In these tasks, humans can use strategies such as adopting the decision of the most confident member of the group to improve their decisions.^24^^,^^30^ However, these are often binary tasks that do not allow studying the question of *how much* social information is integrated in participants’ decisions. This is possible only by measuring continuous quantities, such as numerical estimates or predictions. For these outcomes, the effects of confidence in oneself and others have only been examined in isolation.^15^^,^^18^^,^^19^^,^^20^^,^^21^^,^^25^^,^^26^ Specifically, what remains unclear is how the confidence of self and others interacts when individuals integrate social information, and to what extent the communication of confidence can mitigate the systematic underuse of social information.^31^

Based on the literature summarized above, we expected to observe three main patterns. (1) If individuals regulate information use based on their own internal estimate of reliability, social information use should decrease as a function of participants’ own confidence. (2) If the confidence of the social source serves as a primary cue of credibility of social information, adjustment should increase with others’ confidence. (3) Finally, if individuals integrate self and others’ confidence according to their relative perceived reliability, others’ confidence should have the strongest impact when participants are uncertain, and the social source has high confidence, and should be the lowest when participants’ own confidence is high, and the social source’s is low.

We tested for the presence of these patterns in two pre-registered, incentivized experiments. Participants made quantitative estimates in either an abstract estimation task (Experiment 1) or in a naturalistic task in which they predicted the results of the 2020 USA elections (Experiment 2). After providing an initial estimate and their own confidence in it, they viewed another participant’s estimate and confidence level, then had the opportunity to revise their initial response. Both experiments elicited estimates on continuous scales to provide opportunities for different degrees of adjustments. No feedback was provided about the individual or others’ performance throughout the task. The experiments were similar in structure, with Experiment 2 aiming to achieve high external validity by eliciting predictions about a topic of global relevance at the time of data collection (the 2020 USA election). To isolate the effects of confidence, we controlled for the distance between participants’ own estimates and those they observed, which is known to affect social information use (STAR Methods;^27^^,^^32^).

To clarify the mechanisms that regulate social information use, we focused on two complementary behavioral outcomes. First, we examined differences in the average magnitude of adjustments, that is, how much participants updated their estimates toward those of the social source. This continuous measure captures the degree of social information use more precisely than binary choice tasks, in which individuals either adopt or reject the social source’s choice. Moreover, it reflects the decision process in real-world contexts where social information is not simply accepted or ignored but can be partially integrated, for example, when estimating how many people will attend an event, how many calories are in a meal, or what proportion of the population will vote for a certain political party.

Second, following prior theoretical and empirical work.^20^^,^^32^^,^^33^^,^^34^ We classified participants’ behavior into three strategies: “copy” social information, “stay” with the initial estimate, or “compromise” between the two.^32^ We use the term *heuristics* to refer to these strategies to highlight that they represent qualitatively distinct behavioral responses that provide additional insight into participants’ decision-making processes. Such strategies have been shown to influence belief dynamics in groups and the spread of behaviors across social networks.^6^^,^^20^^,^^32^ We tested how varying levels of self- and other-confidence affected the use of these heuristic strategies.

We expected participants’ responses to depend both on their own and others’ confidence to decide how much social information to use. Specifically, we expected the impact of others’ confidence to be higher when an individual’s own confidence is low. Conversely, when confident, individuals should put less weight on social information regardless of the confidence associated with it. For heuristic strategies, we expected an increase in the proportion of “compromise” and “copy” decisions in rounds where participants’ confidence was low and the confidence of the source was high.

Our main results are consistent across both experiments: self and others’ confidence interact in influencing social information use. Participants generally exhibited egocentric discounting, except when their own confidence was low, and others’ confidence was high. This interaction was evident in both the magnitude of average adjustments and in the frequency of heuristic strategies. When others’ confidence was high, and their own confidence was low, participants were less likely to stick with their own estimate and were more likely to compromise or copy social information. Our study provides convergent evidence that confident sources are heeded more, especially when individuals lack confidence in their own beliefs. We discuss how unconfident individuals face a trade-off between their need for additional information and being vulnerable to those who strategically project high confidence to exert more influence.

## Results

### General setup

**Figure 1:**
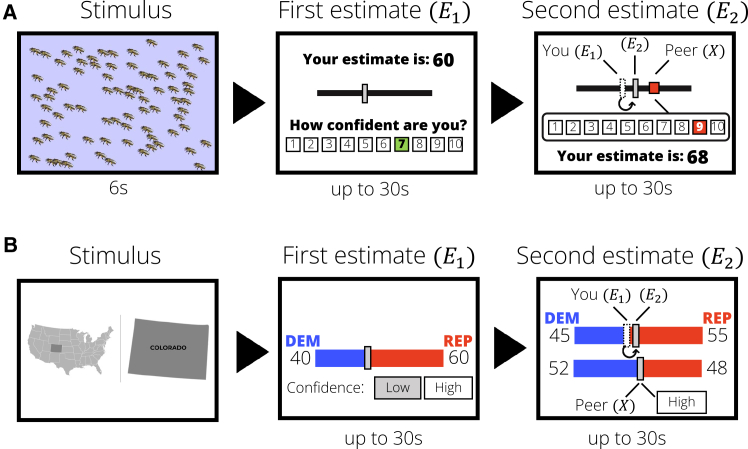
Experimental designs (A) A round of the estimation task (Experiment 1). Participants first observe an image with animals on them for 6s, provide their first estimate of the number (*E*_1_, gray rectangle), and rate their confidence in their estimate on a 1–10 scale. On the next screen, participants observe the estimate of a peer (*X*, red square), the peer’s confidence (highlighted on the scale), and their own first estimate (*E*_1_, white rectangle). Then they provide a second estimate (*E*_2_, gray rectangle). (B) A round of the election prediction task (Experiment 2). For a USA state, participants predict the proportion of Democratic and Republican votes (out of 100; *E*_1_), and rate their confidence as “Low” or “High.” On the next screen, they observe the prediction of a peer (*X*) and their confidence, and make a second prediction (*E*_2_). In both experiments, we measure social information use (s) as the relative adjustment from first to second estimates s=(*E*_2_-*E*_1_)/(*X*−*E*_1_). When personal and social information are weighted equally, *s* = 0.5. In addition to measuring adjustments in each round, we considered three heuristic strategies: “stay” (ignore social information; *s* = 0), “compromise” (move toward social information, 0 < *s* < 1), and “copy” (adopt social information, *s* = 1).

Both experiments involve decision-making tasks in which participants made numerical estimates and were rewarded for accuracy. In Experiment 1, they estimated the number of animals shown in an image (Figure 1A). In Experiment 2, they predicted the proportion of Democratic and Republican voters (out of 100) by state for the 2020 USA presidential elections (Figure 1A). After providing a first estimate and their confidence rating, participants observed the estimate of another participant (a “peer”) and the peer’s confidence rating. Then, they provided a second estimate. We measure social information use (s) as the relative adjustment from the first to the second estimates (see Figure 1 for a formal definition). Our setup isolates the effects of peers’ confidence ratings on social information use, excluding effects of participants’ knowledge of their peers’ relative performance,^35^^,^^36^ differential access to evidence,^25^ or interdependence of payoffs with others.^25^^,^^37^ Furthermore, participants only received feedback about their accuracy after completing all trials of the task, to minimize any learning about their own performance and the possible value of social information.

#### Experiment 1

Participants completed 20 rounds of a validated judgment task^10^ in which they were briefly shown images, and had to estimate the number of animals displayed in them (Figure 1A). To manipulate participants’ own confidence, the image was partly covered in half of the rounds (see STAR Methods). Participants’ adjustments were smaller in rounds where the pictures were fully uncovered (b = −0.05, 95% C.I. = [−0.08, −0.01], see Table S11 for full results of Equation 3). Confidence ratings (1–10) were not incentivized, and as per our pre-registration, they entered the analyses as Low if < 4 and High if > 6 (STAR Methods). This dichotomization allowed for an easier comparison with Experiment 2, and results are robust to treating confidence as an ordinal variable (see Supplemental Items Table S9) and including filler rounds (Supplemental Items Table S10).

#### Experiment 2

Participants completed 20 rounds of a novel, naturalistic prediction task (Figure 1B). In each round, they were asked to predict the outcome of the 2020 USA presidential election for one state (see Table S1 for the full list). Confidence ratings (“Low” or “High”) were incentivized by telling participants that rounds in which they expressed high confidence would be twice as likely to be selected for payment as rounds with low confidence.

### Regression analyses

**Figure 2:**
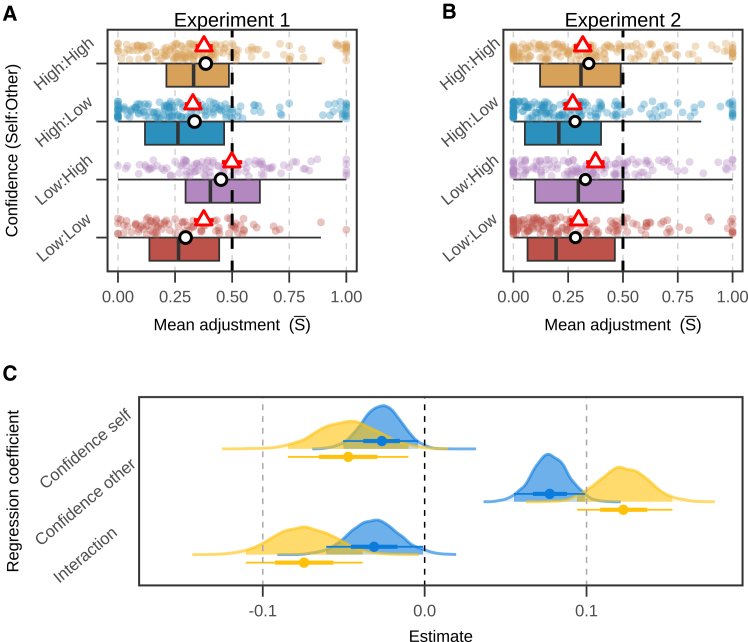
Confidence and social information use (A and B) Individual averages from behavioral experiments. Half boxplots represent the distribution of individual participants’ mean adjustments ($\bar{S}$) by experimental condition (Y axis labels) in each of the experiments. Values for individual participants are plotted as colored dots. White dots indicate the mean of the distribution. Red triangles plot the predicted mean response and standard errors based on conditional effects estimated from the regression model. Black vertical bars in the boxplot show the median. Dashed black vertical lines indicate the point of equal weighting of personal and social information ($\bar{S}$ = 0.5). Values of $\bar{S}$ were mostly below 0.5, even in the Low:High condition (purple dots). For further details on adjustments in individual rounds, see Figure 3. (C) Regression analysis. Posterior distributions of regression coefficients estimated with a Bayesian linear mixed model with “participant” as a random intercept (see Tables S2 and S3 for full model specification and robustness checks). Yellow and blue distributions represent the posterior distribution of estimated coefficients for experiments 1 and 2, respectively. The solid dots and error bars underneath indicate the mean of the distribution, 50% and 95% intervals. The highlighted areas under the curves indicate 95% posterior credible intervals.

We fitted a linear regression with random intercepts for individual participants, setting low confidence (of both self and others) as the reference level to test for the effect of changes in levels of confidence on social information use. In both experiments, adjustments varied substantially with experimental conditions (Figures 2A and 2B).

#### People prioritize personal over social information

Average adjustments in the direction of social information were of $\bar{S}$ = 0.37 in Experiment 1, and $\bar{S}$ = 0.30 in Experiment 2. This magnitude of adjustments is consistent with previous studies using comparable designs (first estimate, then revise.^8^^,^^10^^,^^38^ In both experiments, participants’ average adjustments rarely exceeded $\bar{S}$ = 0.50. This result is indicative of the widely documented phenomenon of egocentric discounting, in which people assign more weight to their own beliefs than to social information, often producing less accurate estimates and receiving smaller monetary payoffs.^11^^,^^31^

#### Individuals respond the most to others’ confidence when their confidence is low

When participants’ own confidence was high, the magnitude of adjustments decreased relative to the reference (Experiment 1: $b_{self}$ = −0.05, 95% C.I. = [−0.08, −0.01], ER > 100, PP = 0.99; Experiment 2: $b_{self}$ = −0.03, 95% C.I. = [−0.05, −0.01], ER > 100, PP = 0.99). This indicates that in rounds where participants were more confident, they were less willing to integrate social information into their beliefs. In rounds where peer confidence was high, adjustment magnitude increased (Experiment 1: $b_{other}$ = 0.12, 95% C.I. = [0.09, 0.15], ER > 100, PP = 1.00; Experiment 2: $b_{other}$ = 0.08, 95% C.I. = [0.06, 0.10], ER > 100, PP = 1.00; Figure 2A). This indicates that seeing a confident peer increased participants’ reliance on social information. Participants’ own confidence and the confidence of the peer credibly interacted with each other (Experiment 1: $b_{self*other}$ = −0.07, 95% C.I. = [−0.11, −0.04], ER > 100, PP = 1.00; Experiment 2: $b_{self*other}$ = −0.03, 95% C.I. = [−0.06, −0.01], ER = 45.78, PP = 0.98; Figure 2A). In both Experiments, the impact of peer confidence on social information was most pronounced when participants’ own confidence was low (Experiment 1: $b_{other}$ = 0.12 [0.09, 0.15] > $b_{other}+b_{self*other}$ = 0.05 [0.03, 0.07], ER > 100, PP = 1.00; Experiment 2: $b_{other}$ = 0.08 [0.06, 0.10] > $b_{other}+b_{self*other}$ = 0.05 [0.03, 0.06]; ER > 100, PP = 1.00), resulting in the highest values of $\bar{S}$ (Experiment 1 $S_{LH}$ = 0.50; Experiment 2: $S_{LH}$ = 0.37).

In Experiment 2, the effects of self- and other confidence were robust to the inclusion of additional pre-registered predictors (Equation 4). Consistent with our expectations, adjustments were larger when peers’ estimates aligned with the same majority as participants (b = 0.03, 95% CI [0.01, 0.04], ER > 100, PP = 1.00), and were smaller for more recognizable States (b = −0.01, 95% CI [−0.02, 0.00], ER > 100, pp = 1.00). Contrary to expectations, adjustments were not credibly affected by participants’ self-reported expertise (b = −0.01, 95% CI [−0.12, 0.10], ER = 1.27, PP = 0.56) or by whether others’ prediction favored their own political orientation (b = 0.01, 95% CI [−0.01, 0.02], ER = 2.95, PP = 0.75).

#### The interaction of one's own and peer confidence influences the use of heuristic strategies

**Figure 3:**
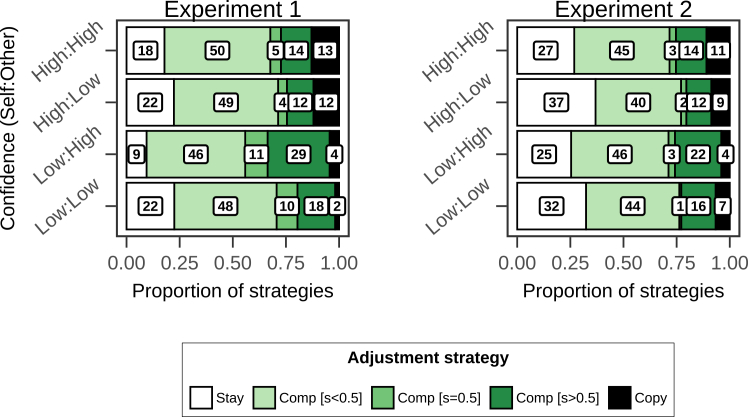
Frequencies of heuristic strategies Bars show the relative frequencies of staying, compromising, and copying, broken down by experimental condition. Numbers in white boxes indicate the percentage of cases in each bin. Instances of “compromise” (green bars) are further broken down into cases where participants gave more weight to personal information (s < 0.5, light green), equal weight to personal and social information (s = 0.5, intermediate green), or more weight to social information (s > 0.5; dark green). For an overview of the strategies broken down by participant, see Figure S1.

Figure 3 shows the distribution of heuristic strategies participants used in individual rounds, broken down by experiment and conditions. Overall, “compromise” was most frequent (Experiment 1: 72%, Experiment 2: 60% of cases), followed by “stay” (19% and 30%), while “copy” was rare (9% and 8%, with most copying done by a small subgroup of participants (see Figure S1). Across conditions, the majority of compromising cases were 0 < s < 0.5 (Figure 3, light green), implying that participants discounted social information relative to their own estimate. Notably, the largest proportion of rounds in which either S = 0.50 or S > 0.50 is observed in the Low:High condition.

We examined differences in proportions of participants’ use of heuristic strategies with a multinomial logistic regression with random intercept for participants and “stay” in the Low:Low confidence condition as the reference level. Across both experiments, the effect of others’ confidence was most pronounced when participants’ own confidence was low. The probability of both compromising (Experiment 1: $b_{self*other}$ = 1.43, 95% CI [0.99, 1.88], ER > 100, PP > 0.99; Experiment 2: $b_{self*other}$ = 0.88, 95% CI [0.55, 1.21], ER > 100, PP > 0.99) and copying (Experiment 1: $b_{self*other}$ = 2.71, 95% CI [1.65, 3.86], ER > 100, PP > 0.99; Experiment 2: $b_{self*other}$ = 1.41, 95% CI [0.59, 2.23], ER > 100, PP > 0.99) was credibly higher in the Low:High condition relative to the baseline. Overall, these results indicate that people are most likely to change their mind when their own confidence is low and the confidence of their social source is high, with an increase toward both “compromise” and “copy” strategies, and a reduction of “stay” (for a full overview of the regression results, see Supplemental Items Tables S4–S8).

## Discussion

In this paper, we examined the joint impact of individuals’ own confidence and peers’ confidence on social information use. Social information use was measured through two behavioral outcomes: (1) the magnitude of adjustments, indicating the extent to which participants integrated others’ estimates into their own, and (2) heuristic strategies, reflecting the frequency of distinct response categories: “stay,” “compromise,” and “copy.” The results from two experiments provide consistent evidence that self and others’ confidence interact in shaping social information use. We discuss these results in the following paragraphs.

Our first result is that across two experiments, the confidence of others had the strongest influence on social information use when participants’ own confidence was low. Given that participants in our study had no information about others’ accuracy, this result suggests that individuals tended to give others’ confidence more weight when uncertain about their own estimates. In everyday life, those seeking to increase their influence on unconfident others can take advantage of this asymmetry by projecting high confidence.^39^ Such behavior can be particularly harmful in online environments, where individuals typically take little time to process information and often use contextual cues (e.g., number of likes and reposts) to evaluate its quality.^40^ In these contexts, confidence signals are not necessarily correlated with accuracy, increasing the risk of misinformation spread and misplaced trust in overconfident sources.

One approach to counter this tendency is metacognitive interventions, i.e., training programs designed to help individuals align their confidence more effectively with their performance.^14^^,^^24^^,^^41^ While some studies have shown encouraging results both in the lab^42^ and outside of it,^43^^,^^44^ others highlighted methodological limitations^45^ and suggest that feedback alone may not be sufficient to improve metacognitive accuracy.^46^ Thus, more work is needed to assess exactly at what stage of the decision process training needs to be delivered. Our results suggest that informing participants about their over-reliance on others’ confidence when their own confidence is low, or informing them even though their confidence is low their estimate is correct, may reduce the over-use of potentially confounding information, thereby promoting more accurate information weighting.

At the same time, when participants were confident, they had less regard for the estimates of others, and less sensitivity to their confidence (Figures 2A and 2C). This finding might reflect participants being “cognitive misers”^47^ saving effort by ignoring social information when it is not needed. Indeed, reduced uncertainty has been associated with less willingness to invest effort to acquire or process social information.^17^^,^^48^^,^^49^^,^^50^ This finding also suggests that convincing confident people requires not only expressing high confidence, but also undermining their confidence in their beliefs. Spreading doubt and questioning scientific consensus are key elements of misinformation campaigns, and strengthening people’s own beliefs may be an effective protection against “merchants of doubt.”^51^^,^^52^

The interaction of one's own and others’ confidence produced consistent effects across the two experiments on heuristic strategies (Figure 3). When one’s own confidence was low, and other’s confidence was high, people were less likely to stay with their initial beliefs and more willing to compromise between their beliefs and social information. When groups have to solve problems that require coordination (e.g., designing policies to curb carbon emissions or preventing pandemics), efficiently reaching an agreement becomes key. In these scenarios, compromising can foster consensus-building,^32^ and our results suggest that confident individuals may accelerate this process by prompting undecided peers to change their minds. Future research could test this hypothesis in collective decision-making experiments by placing confident individuals in strategic positions and measuring their influence on the decision process.

In both experiments, and across conditions, participants consistently assigned more weight to their own estimates than to those of others ($\bar{S}$ < 0.5; Figures 2A and 2B), thus exhibiting egocentric discounting.^11^^,^^20^^,^^31^^,^^38^ While this result could be interpreted as individuals not adopting others’ opinions, but at best meeting in the middle*,* the effects we observe in conditions where others have high confidence are consistently stronger than those normally observed in other comparable advice-taking tasks.^8^^,^^10^^,^^32^ Overall, we conclude that even though egocentric discounting is not fully erased, some of its presence may be explained by participants needing additional context about their social sources. When such context is provided, in this case, confidence, social transmission is facilitated, and social information waste is mitigated.

### Limitations of the study

Our experimental design is not without limitations. First, participants did not receive any feedback on their own performance or that of others. They could therefore not learn how accurate their estimates were. This mimics decision situations that usually lack objective information about the accuracy of beliefs, e.g., in courtrooms^53^ on stock exchanges,^23^^,^^54^ or on social media.^55^ Future studies may assess if the observed disproportionate influence of confident peers holds when people can learn how good they are at a given task (e.g., through recognition by colleagues or using feedback from fact-checking sources such as https://www.factcheck.org/, https://www.politifact.com/). Second, our study builds on the assumption that participants believe that peers use the confidence scale in the same way as they do. Addressing potential differences in how individuals use self-report scales was beyond the scope of this study. Here, we tested how people use information about others’ confidence to update their decision, regardless of whether they believe that those people communicate confidence the same way as they do. Explicitly integrating those individual differences when studying social information use is an interesting direction for future studies building on this work. Another important consideration for future work is to consider a broader population. Our sample was recruited from a WEIRD population, and whether the effects would generalize to other cultures remains an open question. For example, members of individualistic cultures have been shown to be more confident than those of collectivist ones.^56^ Furthermore, it would be interesting to investigate how additional social cues, such as expertise or prestige^57^^,^^58^^,^^59^ interact with confidence. For example, are people more or less responsive to others’ confidence when they can learn about their success or social status? And more, would unconfident experts be followed less than confident laymen?

### Conclusions

Our findings highlight two important considerations for everyday decision-making. First, trusting confident others may be justified in cooperative environments, where learners and sources share common goals (e.g., a hiring committee trying to select the best applicant), but leaves individuals vulnerable to those who falsely project confidence (e.g., social media influencers trying to sell sponsored products^25^^,^^37^). Second, using confidence as a proxy of reliable information assumes that our own, but also others’, confidence is well calibrated and communicated. This is especially important for the communication of information on complex issues, such as climate change, where experts may be more reluctant to express full confidence on a given solution whereas those with a superficial understanding may be convinced of their position.^60^ Thus, when individuals are uncertain and are tempted to use social information from confident others, they should carefully consider their source’s interests,^40^ and fact-check the validity of their source’s claims.

## Resource availability

### Lead contact

Requests for further information and resources should be directed to and will be fulfilled by the lead contact, Andrea Gradassi (andrea.gradassi@gmail.com).

### Materials availability

No materials were newly generated for this paper.

### Data and code availability

The data and code used in this study have been deposited at https://osf.io/38zsy/ and are publicly available as of the date of publication. A live repository is also accessible at https://github.com/AndGrad/Confidence-Self-Others/.

## Acknowledgments

We thank Mubashir Sultan for designing the stimuli and for helping pilot the behavioral task for Experiment 2, Dominik Deffner for the discussion on how to improve the analysis of the data, and all the members of the Connected Minds Lab for providing feedback at various steps of this study. The research was supported by the following grants: 10.13039/501100019541Amsterdam Brain and Cognition Project grant 2018, awarded to LM. A 10.13039/501100003986Jacobs Foundation grant, 10.13039/501100000781European Research Council grant (ERC-2018-StG-803338, WB), 10.13039/501100003246Netherlands Organisation for Scientific Research grant (NWO-VIDI 016.Vidi.185.068), all awarded to WB.

## Author contributions

Conceptualization: A.G, L.M., and W.v.d.B.; methodology A.G, L.M., and W.v.d.B.; investigation: A.G.; formal analysis: A.G, L.M., and W.v.d.B.; writing – original draft Preparation: A.G.; writing – review and editing: A.G, L.M., and W.v.d.B.; supervision: L.M. and W.v.d.B.; project administration: A.G.; funding acquisition: L.M. and W.v.d.B.

## Declaration of interests

The authors declare no conflict of interest.

## Declaration of generative AI and AI-assisted technologies in the writing process

During the preparation of this work, the authors did not use any generative or AI-assisted technologies.

## STAR★Methods

### Key resources table

**Table:** 

REAGENT or RESOURCE	SOURCE	IDENTIFIER
**Deposited data**		

Raw data	This paper	https://doi.org/10.17605/OSF.IO/38ZSY

**Software and algorithms**		

Analysis code	This paper	https://doi.org/10.17605/OSF.IO/38ZSY
R statistical computing environment	https://www.r-project.org/	RRID: SCR_001905
tidyverse	https://cran.r-project.org/web/packages/tidyverse	RRID:SCR_019186
brms	https://cran.r-project.org/package=brms	RRID: SCR_023862
bayestestR	https://cran.r-project.org/package=bayestestR;	N/A
gghalves	https://cran.r-project.org/package=gghalves	N/A
ggthemes	https://cran.r-project.org/package=ggthemes	N/A
sjPlot	https://cran.r-project.org/package=sjPlot;	N/A
cowplot	https://cran.r-project.org/package=cowplot	N/A
ggh4x	https://cran.r-project.org/package=ggh4x	N/A
grid	Base R package; https://www.r-project.org/	N/A
broom	https://cran.r-project.org/package=broom	N/A
pacman	https://cran.r-project.org/package=pacman	N/A
renv	https://cran.r-project.org/package=renv	N/A
here	https://cran.r-project.org/package=here	N/A

### Experimental model and study participant details

#### Human participants

##### Experiment 1

203 participants (mean age = 37.87, s.d = 10.85, range = 22-74, 34.50% female) were recruited for study 1. They were compensated with a fixed fee of $2.00, plus an additional bonus up to $1.00 (average = $0.52, SD = $0.20) depending on their performance, for a total expected payment of ∼ $2.50 (∼$10.00/hr). The bonus was calculated by randomly selecting one of the 20 rounds, and evaluating participants’ performance for that specific round (for each unit their estimate was off, we subtracted $0.05 from their bonus of $1.00, the bonus could not become negative). This setup incentivized participants to invest an equal amount of effort in each round and avoided income effects.

##### Experiment 2

213 participants (mean age = 38.84, s.d. = 11.70, range = 20-71, 45.54% female) were recruited for study 2. Data was collected during the week prior to the 2020 USA presidential election (October 26th-29th 2020). As the experiment revolved around estimating the outcome of USA presidential elections (at the state-level), we restricted the sample to USA residents. Participants were recruited online. Compensation consisted of a fixed fee of $1.70, plus an additional bonus up to $2.00 (average = $0.94, SD = $0.60) depending on the performance, for a total expected payment of ∼ $2.70 (∼ $12.00/hr). The bonus was calculated by randomly selecting two out of the 20 rounds, and evaluating participants’ predictions for those rounds. For each selected round, for each vote their prediction was off the actual election result, we subtracted $0.05 from their bonus; the bonus for a round could not become negative. In this way, we incentivized participants to invest an equal amount of effort in each round and avoided income effects. Bonuses were paid out once the final election results could be estimated with a 1% margin of error.

### Method details

#### Task design

##### Experiment 1

The experiment consisted of a short (15-minute) decision-making task (Figure 1A). In each of the 20 rounds, participants viewed for 6 seconds an image depicting between 50 and 100 animals. The image was either fully revealed (complete information treatment) or partially revealed (uncertain treatment). The aim of this first manipulation was to vary participants’ baseline subjective confidence in their own estimate. After the image had disappeared, participants were asked to give an estimate of the correct number of animals by using a slider, and to rate their confidence in their estimate on a scale from 1 (“Not confident at all”) to 10 (“Extremely confident”). Participants subsequently observed the estimate of a previous participant (a “peer”), who saw the same stimulus for the same amount of time, and had the same task of estimating the number of animals on it. On the same screen, participants observed the peer’s confidence rating, expressed on the same scale as the one that participants used.

This information (estimate and confidence rating) was collected in an earlier iteration of the experiment, where N = 62 participants played the solo condition and after each round reported their confidence ratings. Thus, all the social information displayed was real. From this pool of pre-recorded data, we selected in each round the estimate of a peer that was closest to the target value X’. We defined X’ such that it always pointed in the direction of the true value. Specifically, we defined X'= E_1_ · (1+/-Δ), where E_1_ is the participants initial estimate, where Δ=0.2 if E_1_ was lower than the true value, and Δ=-0.2, if E_1_ was higher than the true value.^10^ If E_1_ was equal to the true value, either value of Δ was chosen with equal probability. Δ was selected to leave participants enough room to adjust, and to control for distance effects that might influence adjustments.^32^^,^^38^

For a peer estimate to be selected as social information, we applied one additional criterion: it had to be associated with a confidence rating that matched the experimental condition of the current round. The ratings were distributed on a scale from 1 (Not confident at all) to 10 (Extremely confident). Three experimental conditions were derived from these ratings: low peer confidence (ratings between 1 and 4), medium peer confidence (ratings between 5 and 6) and high peer confidence (ratings between 7 and 10). These cut-off points were chosen to create a separation between the two conditions of interest, namely low peer confidence and high peer confidence (see pre-registered design).

Over the 20 rounds, participants saw social information reflecting low confidence (between 1 and 4) in 8 rounds, social information reflecting high confidence (between 7 and 10) in 8 rounds, and social information reflecting medium confidence (between 5 and 6) in 4 rounds. We used the medium confidence rounds as “filler” rounds, i.e. rounds that made the experiment more realistic from the participants’ point of view, by including more values from the confidence scale, but which were excluded from some of the analyses (see below). Δ was kept constant at 0.2 in “filler” rounds as well. For this reason, we included fewer medium confidence rounds.

In Experiment 1, as pre-registered, confidence ratings of peers were grouped by categories (Low: 1-4; Medium: 5-6; High: 7-10 on a 10-point scale), and filler rounds in which confidence was “Medium” were excluded from the analysis (see robustness checks analyses analyses in which confidence is treated as ordinal variable and filler rounds are included; cf. Primary Regressions, Supplemental Items Tables S9 and S10).

##### Experiment 2

The experiment consisted in a short (15–20 min) prediction task. Participants had to predict the election outcome for a sample of 20 USA states (see Table S1 for a complete list). Participants played 20 rounds, and in each they had to predict the percentage of votes for Democrats and Republicans for one State. The question was phrased as follows: “out of 100 voters from [state name], how many will vote Democrat or Republican?” First, participants completed a “solo condition”, in which they were prompted to provide a first prediction by using a slider, and rated their confidence in it by clicking on one of two buttons, “Low” or “High”. Participants then moved to the social information part of the experiment, where they could see: (1) the prediction of a previous participant in the experiment for that same state; (2) the confidence rating of the same peer for that round (“Low” or “High”; Figure 1B).

This information (estimate and confidence rating) was drawn from a previous sample of N = 100 participants who completed the same task without social information. As in Experiment 1, the target social information X' was defined X'= E_1_ · (1+/-Δ), where E_1_ is the participants initial estimate. Different from Experiment 1, Δ was not constant, but ranged between .10–.19. Such range still gave participants enough room to adjust, and allowed us to preserve a realistic and varied range of stimuli. The final distribution of target values (e.g. how many rounds had a target at 7 pp., 9.pp away, etc.) was identical for each participant. Finally, to enhance believability, in the two remaining (filler) rounds, social information was either very close or very far from the participant’s first prediction.

### Quantification and statistical analysis

All the analyses were performed using the statistical software *R,*^61^( in the IDE *RStudio.*^62^ Data cleaning and wrangling were supported by the *tidyverse* package,^63^ while Bayesian regression models were fitted using *brms.*^64^ For a full list of packages used in data analysis, see the key resources table).

#### Data cleaning

As pre-registered, and consistent with previous studies using similar paradigms,^10^^,^^32^ statistical models fitted to *s* only included rounds in which the second estimates were a weighted average of own first estimates and social information (that is, 0 ≤ *s* ≤ 1), which were most rounds (92% in Experiment 1, 84% in Experiment 2). This was done to ensure that we measured the effects of the experimental condition on participants’ integration of social information. Responses of *s* < 0 and *s* > 1 imply that participants were either moving away from it or over-adjusting.

#### Statistical reporting

All statistical analyses were performed using Bayesian regression models. To describe our results, we first report the median and the 95% credible intervals of the posterior distributions of regression weights. To quantify evidence in favor of our hypotheses, we calculated Posterior Probabilities (PP) and Evidence Ratios (ER) using the hypothesis() function in *brms*. This procedure computes the proportion of posterior samples consistent with a directional hypothesis (e.g. that the effect of confidence of self on *s* is negative), providing an estimate of the posterior probability that an effect is positive (or negative). For example, Evidence Ratios larger than 100, indicate a ratio of evidence larger than 100:1 for the tested hypothesis over its alternative. We fitted regression models by sampling regression weights from uniform priors, unless otherwise stated.

#### Primary regressions

To analyse participants’ integration of social information as a function of their own and peers’ confidence and interpret the results consistently across the two studies, we identified two regression models that could be fitted to both datasets. First, we fitted a multi-level linear regression on round-by-round individual adjustment (s) across the four combination of confidence (first value refers to self, shorthands in brackets): Low:Low (LL), Low:High (LH), High:Low, High:High. We also used random intercepts for participants (Equation 1):

(Equation 1) $$s\;∼\;confidence_{self}\;*\;confidence_{other}+\left(1\;|\;ID\right)$$

To ensure that this analytical choice did not affect the interpretation of our results, we ran additional robustness checks for Experiment 1 data, including both a regression in which confidence reports are treated as an ordinal variable, and one that includes filler trials (i.e. trials in which confidence values were between 4-6 and as “Medium”; Supplemental Items Tables S9 and S10).

Second, we sought to understand differences in proportions of heuristic strategies*, namely* “*tay*”*,* “*Copy*” or “*Compromise*”, across rounds. To do so, we fitted a multinomial logistic regression on heuristic strategy, using condition (the four combinations of confidence reported above) as a categorical predictor. We included random intercepts for participants (Equation 2):

(Equation 2) heuristicstrategy∼treatment+(1|ID)

Equations 1 and 2 allow for a clear and separate analysis of the two mechanisms at the core of our study, namely differences in mean adjustments, and differences in frequency of heuristic strategies. As an additional robustness check, we fitted a zero–one-inflated beta (ZOIB) regression, which can capture both probabilities of boundary values (0 and 1) and the continuous values within the (0, 1) range, allowing for testing for these two effects simultaneously (for details, see Supplemental Items Tables S3–S12).

#### Additional pre-registered analyses

Beyond the main regressions, we report additional pre-registered analyses that account for specific features of each experiment that could influence participants’ adjustments.

In Experiment 1, half of the trials presented partially covered images to increase uncertainty about the total number of animals. This manipulation was expected to reduce participants’ confidence in their initial estimates. To test whether stimulus uncertainty moderated the influence of confidence, we fitted an extended version of Equation 1 that included an interaction between stimulus uncertainty (i.e., whether the image was covered or not) and participants’ self-reported confidence. We included random intercepts for participants (Equation 3):

(Equation 3) $$s\;∼\;confidence_{self}*uncertainty+confidence_{self}\;*confidence_{other}+\left(1\;|\;ID\right)$$

In Experiment 2, we included additional variables to control for participants’ political attitude, political knowledge and recognizability of the state. We used the following proxies for these variables: *favor*a*bility*: whether the peer’s prediction aligned with the participant’s political preference (e.g., a Democrat seeing a prediction favoring the Democratic party; *same majority*: whether both the participant and the peer predicted the same party to win, regardless of the predicted vote share; *expertise*: participants’ self-rated knowledge of political topics; *population size*: the population of the state subject of the prediction state, used as a proxy for recognizability and salience. Adding these variables as additional predictors to Equation 1, we get (Equation 4.):

(Equation 4) $$s\;∼\;confidence_{self}\;*confidence_{other}+favourability+same\;majority+expertise+population\;size+\left(1\;|\;ID\right)$$

### Additional resources

Pre-registrations links:

Experiment 1: https://osf.io/xv35k.

Experiment 2: https://osf.io/vksjy.
