# Supplementary material for: Own and others’ confidence in social information use
Source: iScience. 2026 May 22;29(6):115968. doi: 10.1016/j.isci.2026.115968 (PMC13223951; doi:10.1016/j.isci.2026.115968)
Supplement: Document S1. Figures S1–S4, Tables S1–S14, and Methods S1 [file mmc1.pdf]

iScience, Volume *29*

Supplemental information

Own and others' confidence

in social information use

Andrea Gradassi, Wouter van den Bos, and Lucas Molleman

Methods S1: Deviations from pre-registration

Experiment 1

Pre-registration: https://osf.io/xv35k/).
We report the full results testing for the effects of stimulus uncertainty in the Supplementary Files, Table S3.

Experiment 2

In the pre-registered model (https://osf.io/vksjy) confidence of self and others were included as main effects, and not as interactions. This choice was made to make the interpretation of the models with multiple control variables simpler. However, for consistency with Experiment 1, we now include an interaction as well (cf. Equation 1 in the Primary Regression Text, Main Text)

Supplemental Figures

Figure S1. Proportion of strategies use by individual participants.

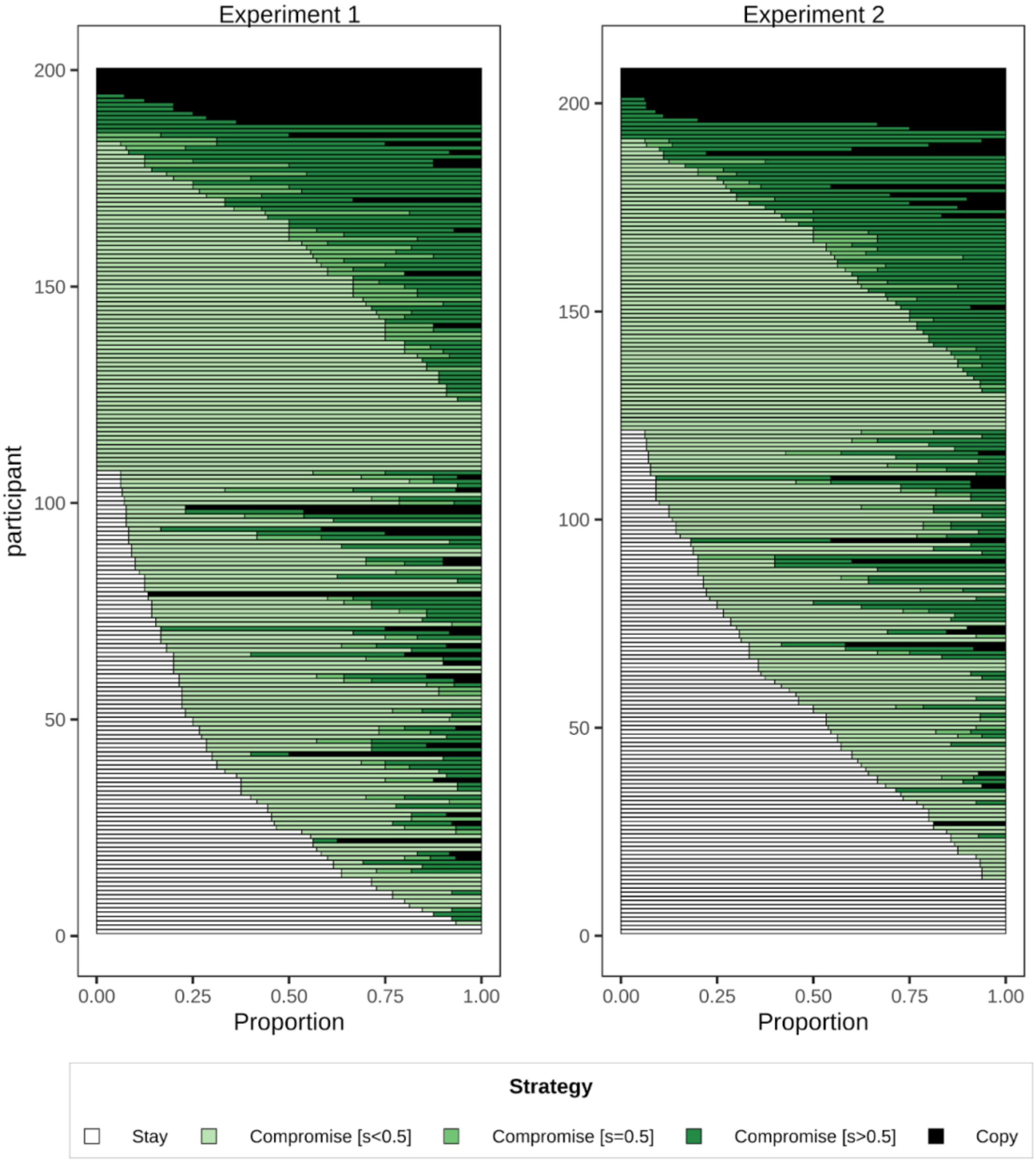

Proportion of each strategy used by individual participants. Most participants deployed a combination of different strategies throughout the experiment; however, we observed a portion of individuals who always chose to 'Copy' (exp1:6 participants (ppts); exp2: 7 ppts), or always who chose to 'Stay; (exp1: 2 ppts, exp2: 13 ppts). While the overall pattern is comparable, in Experiment 2, the frequency of 'Stay' was higher than Experiment 1 (6% vs 3%).

Figure S2. Participants' predictions by State in experiment 2.

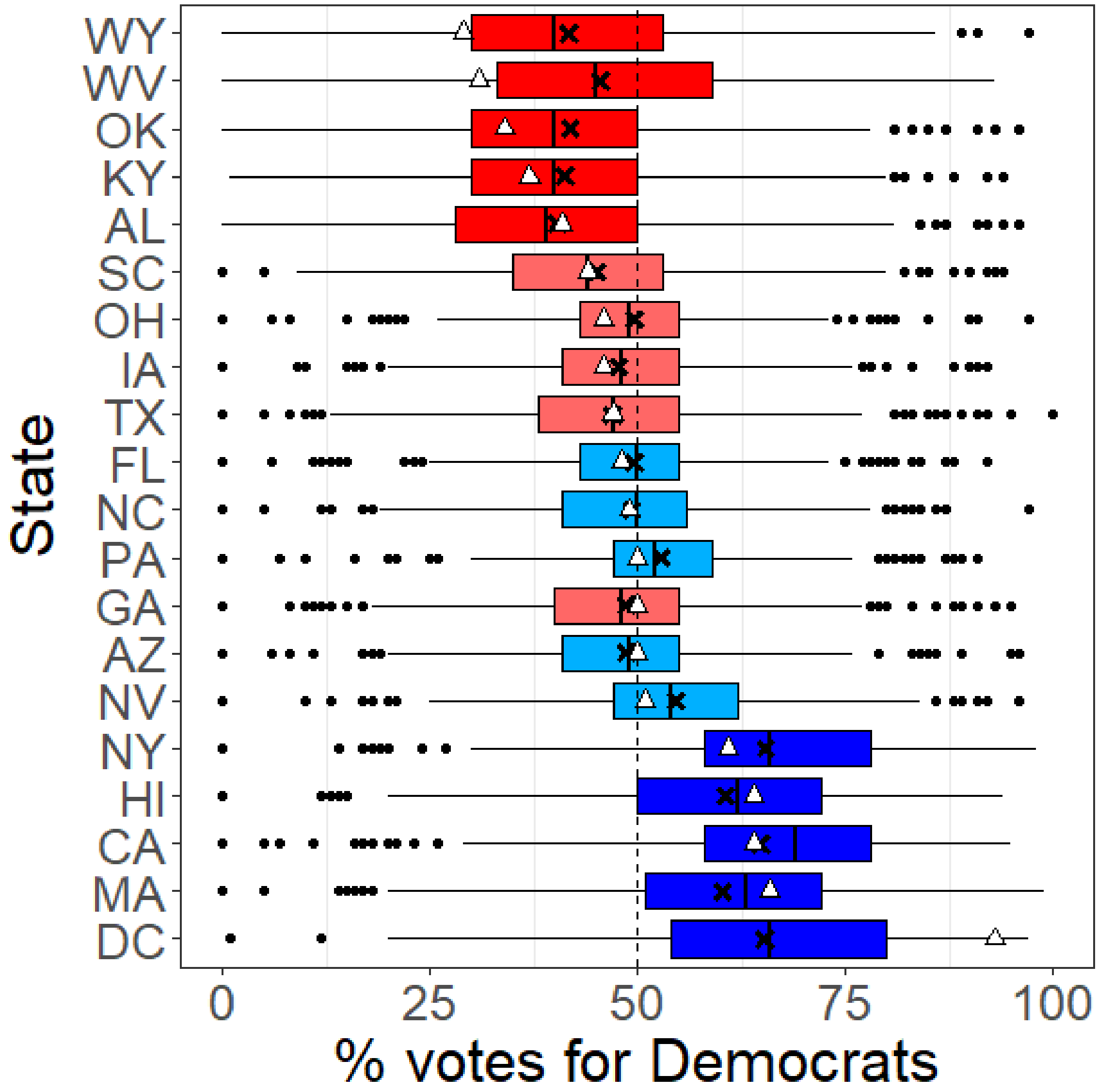

Boxplots represent individual predictions for each State. Black bars represent the median, Black crosses the mean, white filled triangles the real outcome of the elections. Darker colours (ark red; dark blue) indicate states selected in which the poll where less uncertain, while lighter colours (light red; light blue) represent states in which uncertainty was higher. For more information in this distinction, see table S1.

Figure S3 Distribution of s in the excluded trials.

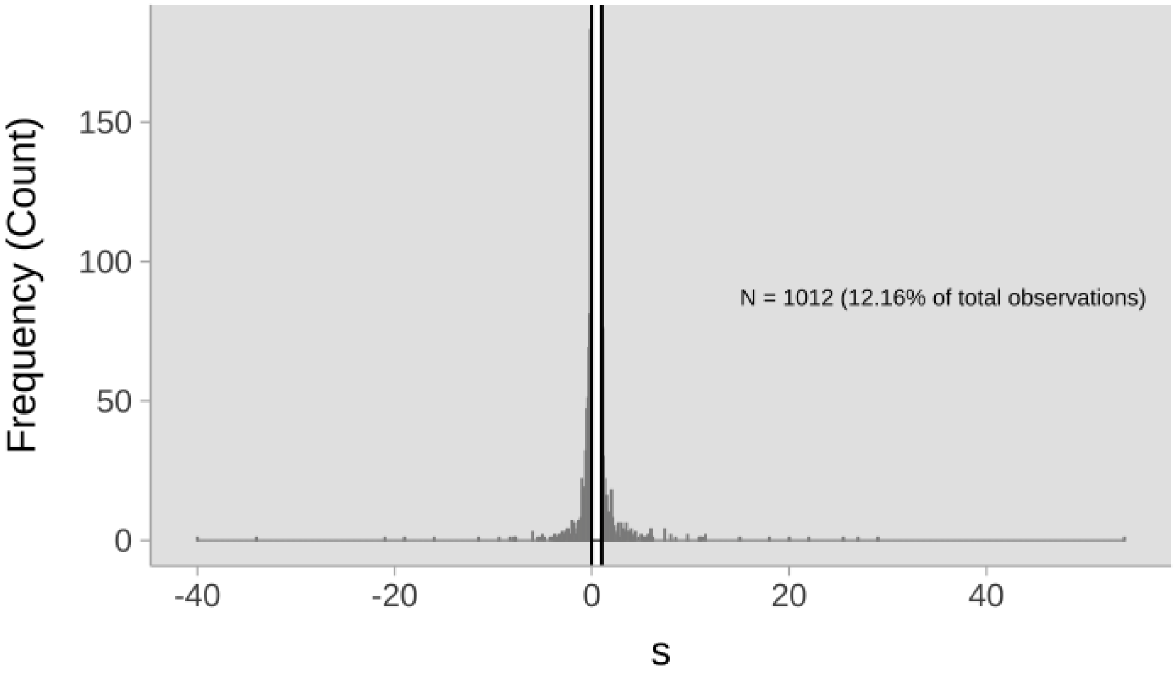

Histogram reporting the distribution of the outcome variable in Experiment 1 for the rounds that were excluded from the analysis. Values of 0>s>1 indicate that participants' second estimate is not a weighted average of their first estimate and social information.

Figure S4. Correct first estimates.

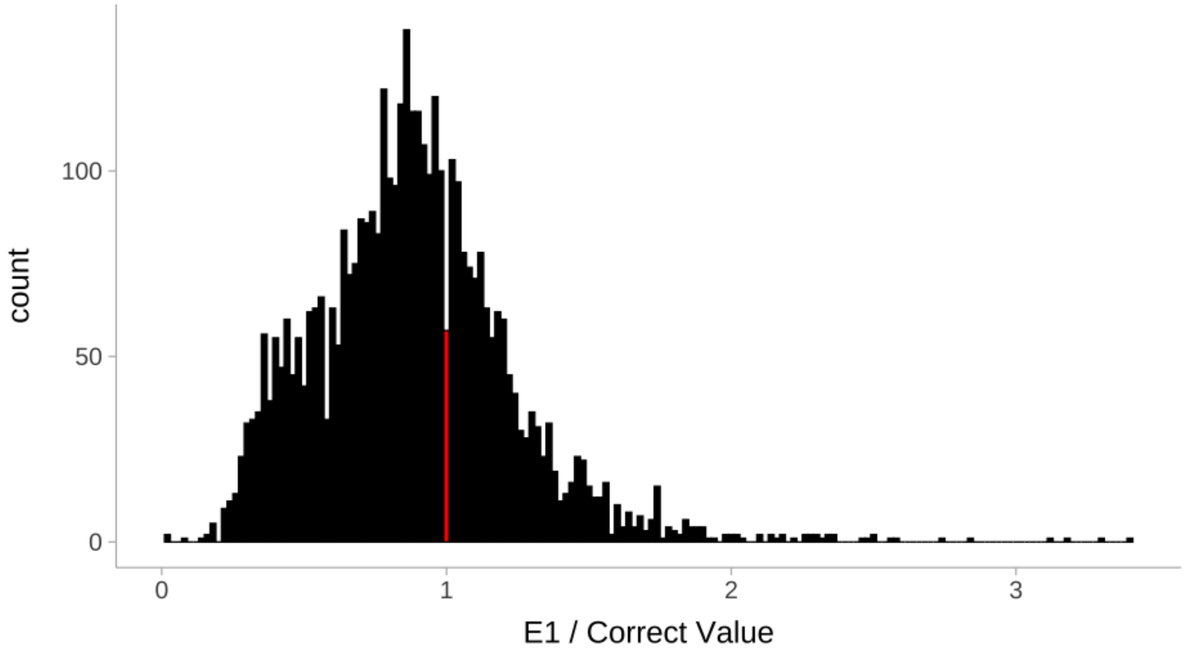

Histogram representing participants' accuracy in their first estimates. The quantity displayed on the x-axis is a ratio of the number entered by participants divided by the correct number. Values smaller than 1 indicate that participants were underestimating the total number of animals, while values larger than 1 indicate an overestimation. Values equal to 1 (N= 54) indicate a correct guess.

Supplemental Tables

Table S1. States used as stimuli in the Election Task.

**State name**	**Predicted winner**	**Uncertainty**
Alabama	Republicans	Low
Arizona	Democrats	High
California	Democrats	Low
Washington D.C.	Democrats	Low
Florida	Democrats	High
Georgia	Republicans	High
Hawaii	Democrats	Low
Iowa	Republicans	High
Kentucky	Republicans	Low
Massachusetts	Democrats	Low
Nevada	Democrats	High
New York	Democrats	Low
North Carolina	Democrats	High
Ohio	Republicans	High
Oklahoma	Republicans	Low
Pennsylvania	Democrats	High
South Carolina	Republicans	High
Texas	Republicans	High
West Virginia	Republicans	Low
Wyoming	Republicans	Low

States were selected to ensure a balance between predicted winners (10 predicted Democrats and 10 predicted Republicans) and uncertainty of the outcome (High or Low), according to polls' predictions made by the specialized website http://fivethirtyeight.com. Predictions were consulted in the month before the election (October 2020).

Table S2. Bayesian linear mixed model Results from Experiment 1.

	Estimate (CI)	Rhat	PP	ESS
Intercept	.38 (.33, .42)	1.00	1.00	777.88
Confidence self: High	-.05, (-.08, -.01)	1.00	0.99	2,988.55
Confidence other: High	.12 (.09, .15)	1.00	1.00	4,310.90
Confidence Self X Confidence Other	-.07 (-.11, -.04)	1.00	1.00	4,301.18

Estimates of a Bayesian linear mixed model with participants as random intercepts fitted to individual rounds adjustments (s). Values between parentheses indicate 95% credible intervals.

Table S3. Bayesian Linear mixed Model Results from Experiment 2 (including robustness checks)

	Estimate (CI)	Rhat	pd	Bulk_ESS	Tail_ESS
Intercept	.31 (.22, .41)	1.00	1.00	591	1131
Confidence self: High	-.03, (-.05, -.00)	1.00	0.99	5466	4239
Confidence other: High	.08 (.06, .10)	1.00	1.00	5083	4384
Confidence Self X Confidence Other	-.03 (-.06, -.00)	1.00	0.56	5048	4739
Robustness checks					
Participant's expertise	-.01 (-.14, .12)	1.01	0.75	595	1059
Desirable outcome: yes	.01 (-.01, .02)	1.00	0.99	8946	4365
Same majority: yes	.03 (.01, .04)	1.00	0.99	8645	4466
Population size	-.01 (-.02, 0.00)	1.00	0.98	5048	3935

Estimates of a Bayesian linear mixed model with participants as random intercepts fitted to individual rounds adjustments (s). Robustness checks were included to test whether participants would be influenced by seeing: 1) social information in line with their political preferences (*Desirable outcome,* i.e. a Democrat seeing a prediction of Democrats winning for that state); 2) social information showed the same winning party, even if the exact percentages differed (*Same majority)*, and 3) states with larger population sizes, as a proxy for states covered more by the press, and thus easier to predict. Including the robustness checks did not change the effects of confidence (both of self and others) on social information use. Values between parentheses indicate 95% credible intervals.

Table S4. Results of Bayesian Multinomial Logistic regression (Experiment 1).

	Estimate (CI)	Rhat	Bulk ESS	Tail ESS
Compromise Intercept	2.26 (1.73, 2.80)	1.00	1320	2710
Copy Intercept	-5.23 (-7.10, -3.59)	1.00	1612	2339
Compromise LH	1.42 (0.91,1.97)	1.00	4179	3776
Compromise HL	-0.56 (-1.08,-0.05)	1.00	2007	2679
Compromise HH	-0.08 (-0.600.43)	1.00	1987	2808
Copy LH	2.71 (1.46,4.08)	1.00	3316	3475
Copy HL	0.43 (-0.99,1.92)	1.00	2047	3008
Copy HH	1.41 (0.02,2.88)	1.00	2028	3026

Estimates of a Bayesian multinomial logistic regression with participants as random intercepts fitted to individual rounds adjustments (s). Values between parentheses indicate 95% credible intervals. For a full breakdown of results, see Table S8.

Table S5. Results of Bayesian Multinomial Logistic regression (Experiment 2).

	Estimate (CI)	Rhat	Bulk ESS	Tail ESS
Compromise Intercept	1.45 (0.90, 2.01)	1.00	942	1634
Copy Intercept	-7.73 (-10.78, -5.44)	1.00	2060	2890
Compromise LH	0.88 (0.49,1.27)	1.00	7246	4651
Compromise HL	-0.24 (-0.63, 0.13)	1.00	5735	5070
Compromise HH	0.64 (0.26,1.02)	1.00	5075	4890
Copy LH	1.41 (0.43, 2.38)	1.00	8272	4969
Copy HL	-0.65 (-1.75, 0.42)	1.00	5628	4800
Copy HH	0.39 (-0.62, 1.39)	1.00	5365	5060

Estimates of a Bayesian multinomial logistic regression with participants as random intercepts fitted to individual rounds adjustments (s). Values between parentheses indicate 95% credible intervals. For a full breakdown of results, see Table S8.

Table S6. Conditional probabilities of adjustment strategies in experiment 1 & 2, without random intercepts.

	Experiment 1			Experiment 2		
Heuristic	Stay	Compromise	Copy	Stay	Compromise	Copy
Treatment						
LL	.22 (.18, .27)	.76 (.71, .80)	.02 (.01, .03)	.32 (.29, .36)	.61 (.57, .65)	.07 (.05, .09)
LH	.09 (.07, .13)	.86 (.82, .89)	.04 (.03, .07)	.25 (.22, .29)	0.71 (.67, .74)	.04 (.02, .06)
HL	.22 (.19, .25)	.66 (.62, .69)	.12 (.10, .15)	.37 (.33, .40)	.54 (.51, .58)	.09 (.07, .11)
HH	.18 (.15, .21)	.69 (.66.72)	.13 (.11, .16)	.27 (.24, .30)	.62 (.59, .65)	0.11 (.09, .13)

Conditional probabilities of each adjustment strategy (Stay, Compromise, Copy) by condition, estimated by a Bayesian Multinomial Logistic regression. Values between parentheses indicate 95% credible intervals. Data reported here matches Figure 3 in the main text.

Table S7. Conditional probabilities of adjustment strategies in experiment 1 & 2, with random intercepts

	Experiment 1			Experiment 2		
Heuristic	Stay	Compromise	Copy	Stay	Compromise	Copy
Treatment						
LL	.09 (.06, .15)	.90 (.85, .94)	.00 (.00, 00)	.19 (.12-28)	.80 (.71, .88)	.00 (.00, 00)
LH	.02 (.01, .04)	.97 (.95, .99)	.00 (.00, 00)	.09 (.5, .14)	.90 (.85, .94)	.00 (.00, 00)
HL	.15 (.10, .22)	.84 (.78, .90)	.00 (.00, 01)	0.23 (.15, .33)	.76 (.66, .85)	.00 (.00, 00)
HH	.10 (.06, .15)	.85 (.85, .93)	.00 (.00, 01)	0.11 (.06, .17)	00.89 (.82, .93)	.00 (.00, 00)

Conditional probabilities of each adjustment strategy (Stay, Compromise, Copy) by condition, estimated by a Bayesian Multinomial Logistic regression with participant ID included as a random intercept. Values between parentheses indicate 95% credible intervals.

Table S8 Bayesian Hypothesis testing of multinomial logistic regression

Hypothesis	Estimate (C.I.)	ER	PP		Experiment
(μ Compromise Intercept) > 0	2.26 (1.83, 2.71)	>100	1.00	*	Experiment 1
(μ Compromise Intercept) > 0	1.45 (0.97, 1.91)	>100	1.00	*	Experiment 2
(μ Compromise interaction HH) > 0	-0.08 (-0.51, 0.35)	0.60	0.37		Experiment 1
(μ Compromise interaction HH) > 0	0.64 (0.32, 0.95)	>100	1.00	*	Experiment 2
(μ Compromise interaction HL) < 0	-0.56 (-1.00, -0.13)	60.22	0.98	*	Experiment 1
(μ Compromise interaction HL) < 0	-0.24 (-0.57, 0.07)	8.24	0.89		Experiment 2
(μ Compromise interaction LH) > 0	1.42 (0.99, 1.88)	>100	1.00	*	Experiment 1
(μ Compromise interaction LH) > 0	0.88 (0.55, 1.21)	>100	1.00	*	Experiment 2
(μ Copy Intercept) < 0	-5.23 (-6.78, -3.84)	>100	1.00	*	Experiment 1
(μ Copy Intercept) < 0	-7.73 (-10.10, -5.77)	>100	1.00	*	Experiment 2
(μ Copy interaction HH) > 0	1.41 (0.23, 2.64)	42.17	0.98	*	Experiment 1
(μ Copy interaction HH) > 0	0.39 (-0.44, 1.21)	3.69	0.79		Experiment 2
(μ Copy interaction HL) < 0	0.43 (-0.78, 1.64)	2.51	0.72		Experiment 1
(μ Copy interaction HL) < 0	-0.65 (-1.57, 0.24)	7.70	0.89		Experiment 2
(μ Copy interaction LH) > 0	2.71 (1.65, 3.86)	>100	1.00	*	Experiment 1
(μ Copy interaction LH) > 0	1.41 (0.59, 2.23)	460.54	1.00	*	Experiment 2

Results of hypothesis testing from the hypothesis() function in brms. The Evidence Ratio (ER) quantifies support for the tested hypothesis relative to the alternative, and the Posterior Probability (Post. Prob.) indicates the probability that the true direction of the effect is consistent with the observed one, given the posterior draws. Reported 95% credible intervals correspond to the 95% posterior credibility intervals.

Table S9. Results of regression analysis with confidence as ordinal variable in experiment 1

	s		
	Estimates	std. Error	CI (95%)
Intercept	0.37	0.02	0.34 – 0.41
Confidence Self (High)	-0.06	0.01	-0.07 – -0.05
Confidence Other (High)	0.03	0.00	0.03 – 0.04
Interaction	-0.01	0.00	-0.02 – -0.01

Estimates of Bayesian linear mixed model with participants as random intercepts fitted to individual rounds adjustments (s), in experiment 1. Contrary to the model reported in the main text, confidence was included as a continuous variable, exploiting the full variation present in the confidence scale (range 1-10). Predictors were standardized before fitting the model for ease of interpretation. The results are qualitatively identical to the model reported in the main text: high confidence of self led to a reduced use of social information, while high confidence of others led to an increase in social information use. Finally, we observe a credible interaction between confidence of self and confidence of others.

Table S10. Results of regression analysis with filler rounds (Medium confidence) experiment 1

	s		
Predictors	Estimates	std. Error	CI (95%)
Intercept	0.38	0.02	0.34 – 0.41
Confidence Self (Medium)	-0.03	0.01	-0.06 – -0.00
Confidence Self (High)	-0.05	0.01	-0.08 – -0.03
Confidence Other (Medium)	0.04	0.02	0.01 – 0.07
Confidence Other (High)	0.11	0.01	0.08 – 0.13
Confidence Self (Medium): Confidence Other (Medium)	-0.01	0.02	-0.05 – 0.03
Confidence Self (High): Confidence Other (Medium)	-0.03	0.02	-0.07 – 0.01
Confidence Self (Medium): Confidence Other (High)	-0.03	0.02	-0.06 – 0.01
Confidence Self (High): Confidence Other (High)	-0.06	0.02	-0.09 – -0.03

Estimates of Bayesian linear mixed model with participants as random intercepts fitted to individual rounds adjustments (s), in experiment 1. Different to the model reported in the main text, here we include filler trials (Medium confidence treatment). The results are consistent with the model reported in the main text, showing that in Medium confidence rounds, participants used less social information than in the Low Confidence rounds, and more than in the High confidence ones.

Table S11. Results of regression analysis of stimulus uncertainty in Experiment 1

	Estimates	std. Error	CI (95%)
Intercept	0.39	0.02	0.35 – 0.44
Uncertainty (Low)	-0.05	0.02	-0.08 – -0.01
Confidence Self (High)	-0.08	0.02	-0.12 – -0.04
Confidence Other (High)	0.12	0.01	0.10 – 0.16
Uncertainty (Low): Confidence Self (High)	0.07	0.02	0.03 – 0.11
Confidence Other (High): Confidence Self (High)	-0.08	0.02	-0.11 – -0.04

Estimates of Bayesian linear mixed model with participants as random intercepts fitted to individual rounds adjustments (s), in experiment 1. Relative to the model in Equation 1 in the main text, here we explicitly model an interaction between participants' own confidence and the uncertainty treatment in which we partially cover the stimulus. The model shows that social information use was lower when stimuli were fully visible (uncertainty = Low) (Estimate = –0.05, 95% CI [–0.08, –0.01]), and when their own confidence was high (Estimate = –0.08, 95% CI [–0.12, –0.04]), but increased when others' confidence was high (Estimate = 0.12, 95% CI [0.10, 0.16]). A significant interaction between uncertainty and self-confidence (Estimate = 0.07, 95% CI [0.03, 0.11]) indicates that the negative effect of self-confidence was reduced when participants were uncertain. Additionally, an interaction between self- and other-confidence (Estimate = –0.08, 95% CI [–0.11, –0.04]) shows that the positive influence of others' confidence weakened when participants were themselves confident.

Table S12. **Regression estimates from the Bayesian zero-one-inflated beta (ZOIB) regression on *s* (Experiment 1)**

	Estimate (95% CI)	Rhat	Bulk ESS	Tail ESS
μ Intercept	-0.47 (-0.61, -0.33)	1.00	2939	5628
φ Intercept	2.16 (1.92, 2.39)	1.00	6751	8532
zoi Intercept	-1.96 (-2.51, -1.43)	1.00	4148	7275
coi Intercept	-4.50 (-6.98, -2.31)	1.00	6489	7189
μ: Confidence Self (High)	-0.10 (-0.24, 0.03)	1.00	7116	8833
***μ:* Confidence Other (High)**	**0.20 (0.09, 0.32)**	1.00	9978	9673
μ: Confidence Self (High) x Confidence Other (High)	-0.13 (-0.27, 0.01)	1.00	10089	9671
φ: Confidence Self (High)	-0.11 (-0.40, 0.17)	1.00	7441	8940
φ: Confidence Other (High)	-0.13 (-0.41, 0.16)	1.00	8114	8744
φ: Confidence Self (High) x Confidence Other (High)	0.27 (-0.07, 0.61)	1.00	8129	9467
**zoi: Confidence Self (High)**	**0.68 (0.19, 1.17)**	1.00	9450	9695
**zoi: Confidence Other (High)**	**-0.98 (-1.44, -0.50)**	1.00	12789	9741
**zoi: Confidence Self (High) x Confidence Other (High)**	**0.68 (0.12, 1.24)**	1.00	12484	9494
coi: Confidence Self (High)	0.26 (-2.19, 2.68)	1.00	6352	7948
**coi: Confidence Other (High)**	**3.40 (1.22, 6.00)**	1.00	8482	6832
coi: Confidence Self (High) x Confidence Other (High)	-2.15 (-4.86, 0.24)	1.00	8353	6610

Estimates from a Bayesian zero-one-inflated beta (ZOIB) regression fitted to individual adjustments (s). The mean (*μ*) component models variation in *s* (weight assigned to social information) as a beta distribution, thus without considering values of 0 and 1 ('Compromise'). φ models the precision of the beta distribution. *zoi* models the 0-1 inflation, i.e. the probability of a binary rating ('Stay' or 'Copy'), and finally, *coi* models conditional one-inflation ('Copy'). Participants relied more on social information when others were highly confident ($\mu = 0.20$, 95% CI [0.09, 0.32]) and slightly less when their own confidence was high ($\mu = -0.10$, 95% CI [–0.24, 0.03]). Interaction effects indicate that participants' weighting of social information depended on the combination of their own and others' confidence. Coi indicates more copying when confidence of others is high ($b = 3.40$, 95% CI [1.22, 6.00]).

Table S13. Regression estimates from the Bayesian zero-one-inflated beta (ZOIB) regression on s (Experiment 2)

Predictor	Estimate (95% CI)	Rhat	Bulk ESS	Tail ESS
μ: Intercept	-0.61 (-0.99, -0.22)	1.00	2243	4180
φ Intercept	1.80 (1.40, 2.19)	1.00	6494	8685
zoi Intercept	-1.11 (-2.51, 0.29)	1.00	1876	3823
coi Intercept	-7.29 (-14.98, -0.50)	1.00	3250	5135
μ: : Confidence Self (High)	-0.11 (-0.24, 0.02)	1.00	7487	8311
***μ:* Confidence (Other: High)**	**0.21 (0.09, 0.33)**	1.00	8384	8530
μ: Expertise	0.01 (-0.57, 0.59)	1.00	2232	4327
μ: Favorability (yes)	0.03 (-0.05, 0.11)	1.00	15347	10109
μ: Same Majority (yes)	0.06 (-0.03, 0.15)	1.00	12395	9167
***μ:* Population**	**-0.05 (-0.09, -0.01)**	1.00	16711	9883
μ: Confidence Self (High) x Confidence Other (High	-0.09 (-0.24, 0.08)	1.00	7566	8154
φ: Confidence Self (High)	0.16 (-0.08, 0.40)	1.00	7823	8225
φ: Confidence (Other: High)	0.02 (-0.21, 0.25)	1.00	8462	9486
φ: Expertise	-0.25 (-0.82, 0.32)	1.00	6161	8322
φ: Favorability (yes)	-0.08 (-0.24, 0.07)	1.00	13327	9540
**φ: Same Majority (Yes)**	**0.28 (0.11, 0.45)**	1.00	12663	9818
φ: Population	0.01 (-0.06, 0.09)	1.00	13679	9693
φ: Confidence Self (High) x Confidence Other (High)	-0.05 (-0.36, 0.26)	1.00	7903	8937
zoi: Confidence Self (High)	0.22 (-0.17, 0.60)	1.00	9879	8968
**zoi: Confidence (Other: High)**	**-0.69 (-1.07, -0.33)**	1.00	10381	8922
zoi: Expertise	0.68 (-1.49, 2.82)	1.00	1749	3168
zoi: Favorability	-0.00 (-0.25, 0.24)	1.00	16538	9489
**zoi: Same Majority**	**-0.61 (-0.88, -0.34)**	1.00	16542	8650
**zoi: Population**	**0.17 (0.05, 0.30)**	1.00	18993	8819
zoi: Confidence Self (High) x Confidence Other (High)	-0.02 (-0.51, 0.49)	1.00	9121	8325
coi: Confidence Self (High)	-1.50 (-3.62, 0.50)	1.00	7980	7933
coi: Confidence Other (High)	0.83 (-1.18, 2.94)	1.00	8269	8202
coi: Expertise	-0.28 (-11.08, 10.82)	1.00	2987	4775
coi: Favorability (yes)	-0.84 (-2.17, 0.40)	1.00	14550	9564
coi: Same Majority (yes)	0.60 (-0.82, 2.02)	1.00	11434	9408
coi: Population	0.02 (-0.48, 0.53)	1.00	18260	8587
coi: Confidence Self (High) x Confidence Other (High)	1.46 (-1.10, 4.09)	1.00	7675	8390

Estimates from a Bayesian zero-one-inflated beta (ZOIB) regression fitted to individual adjustments (s). The mean (μ) component models variation in *s* (weight assigned to social information) as a beta distribution, thus without considering values of 0 and 1 ('Compromise'). φ models the precision of the beta distribution. *zoi* models the 0-1 inflation, i.e. the probability of a binary rating ('Stay' or 'Copy'), and finally *coi* models conditional one-inflation ('Copy'). Higher Confidence of others was associated with increased adjustments (μ = 0.21, 95% CI [0.09, 0.33]), whereas Population size was associated with smaller adjustments. The probability of extreme (0 or 1) responses decreased when confidence of others was high (zoi = –0.69, [–1.07, –0.33]) and when participants and peers predicted the same majority (zoi = –0.61, [–0.88, –0.34]), and increased with Population size (zoi = 0.17, [0.05, 0.30]).

Table S14 Proportion of observations in each condition in each experiment. 2

	Experiment 1	Experiment 2
**condition**	**N of observations**	
LL	91	158
LH	99	143
HL	162	178
HH	163	192

Number of observations in each of the experimental conditions.

§4 Experiment Instructions

Experiment1

Welcome!

In this HIT we ask you to complete a **10-15 minute task**.
In this task, you can earn points.

You will make 2 decisions over multiple rounds.

At the end of the HIT, one decision of one round will be randomly selected for payment.

The number of points you earn in that round will determine your bonus, at the following rate: 100 points = $1.00.

Your bonus will be paid on top of your guaranteed participation fee of **[fee]**

Upon completion, you will receive a unique code to collect your payment on MTurk.

Informed consent:
By checking this box you give us **informed consent** that we can use your answers in anonymized form for research purposes only.

HIT description

In this **HIT** you will make a number of **estimates**.

The number of points you earn depends on **how accurate** your estimates are.

Click continue to proceed to the task instructions. *Please read these instructions carefully.*

This task consists of 20 rounds.

Each round, is divided in two parts: ***part A*** *and* ***part B***.

In **part A** you will observe an image of animals, and you will have to estimate how many animals are in it.
For example:

[Example image]

After **6 seconds**, the image will disappear. You then have to estimate how many animals were displayed.
The closer your estimate is to the actual number of animals, the more points you will earn.

In some rounds the image will be partly hidden, as shown in the example below.

In these rounds, your task will be to estimate the **number of total animals in the underlying - full - image**
(so, your goal is to estimate the number of **ALL animals**, including the hidden ones).

Your will enter your estimates with a slider.
To move the slider handle, you can either drag it with your mouse, click on the slider bar or use the arrow keys.

Note: please enter your estimates within the time limit on your screen.
If you do not make your estimate and press 'Continue' *before the timer reaches zero*, **you will be removed from the HIT** and we **will not be able to pay you!**

Here is an example to make you familiar with the slider:

After choosing a number, you also will be asked to rate **how confident you are in your estimate**, using a scale like this one:

[Example confidence scale]

Once you have reported your confidence for **part A**, **part B** of the round begins.

In **part B**, you will observe the **part A** estimate of another MTurker.
Next to their estimate, you will also see **how confident they were about it.**

Over 200 MTurkers participated in a previous session in which they completed this task.
In each round, you can observe the **part A** estimate of **one** of these previous MTurkers.

In **part B** you have to enter a second estimate.

You can enter the same estimate as in **part A**, or adjust it as you wish.

Once you have entered your **part B** estimate, the round is over and a new round begins.

Your bonus earnings

The more accurate your estimates, the more points you can earn.

At the end of this HIT, the computer will randomly select your **part A** or **part B** estimate of 1 round for payment.
If your estimate in that round was ***exactly right*, you earn 100 points.**
For each number that you are off, we subtract 5 points.
The number of points you earn cannot become negative.

For example, if the actual number of animals in the image was 70, and your estimation was 63, you were 7 off.

This would mean that we subtract 7 *x* 5 = 35 points. Your earnings for that estimate would be 100 - 35 = 65 points.

Click 'Continue' if you understood your task.
A brief quiz will follow to check your understanding.

Experiment 2

Welcome!

We are conducting an online study on the US Presidential Elections of 2020.

In this task, you will be presented with a selection of States and you will have to predict what voters will do.

Informed consent:
By checking this box you give us informed consent that we can use your answers in anonymized form for research purposes only.

The study is split into two general sections:

1. Prediction Task
2. Exit Questions

In the **Prediction Task** you will be asked to predict how many **out of 100 voters** will vote for the **Democratic** or the **Republican** party for a selection of 20 States.

In total, you will play **20 rounds.** Each round is divided in two parts, **part A** and **part B.**

Please note: In this study we only consider the ***Republican*** *and* ***Democratic*** *voters.*
Other parties will get a share of votes, but for simplicity's sake here we only consider the two main Parties.

In **part A** of each round, you will **use a slider** to indicate how many out of 100 voters you think will vote for the **Republican** or the **Democratic** party.

Below, we see a participant entering their prediction for **Colorado**.
They predict **60 out of 100** voters to choose **Democratic**, and the remaining **40** to choose **Republican**.

Dragging the slider handle to the **right** indicates that you expect more **Democrat** voters.

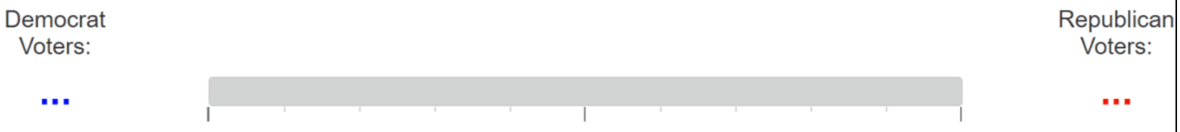

If you expect more **Republican** voters, drag the slider handle to the left.

Please note: *you first have to click on the slider to activate it.*

Press **Next** to try this out.

A bit about money!

At the end of the Task we will randomly select one of your predictions to determine **your bonus prize!**

If this prediction is exactly right, you earn **$1.00**.
For **each number (vote)** you are off, we **subtract $0.05.**

So, the closer you are to the actual outcome, the more money you will earn.

Press **Next** for an example.

Imagine you predicted that in Colorado **67 out of 100** voters will vote **Democrat**, and **33 Republican**.

If the election outcome is that **60% of voters in that state** actually voted **Democrat**, it means you were 67 - 60 = 7 *votes off.*

Therefore, we would subtract 7 x $0.05 = $0.35 from your bonus, and you would earn $0.65. Please note, your bonus cannot become negative. If you are more than 20 votes off, and that round gets randomly selected, your bonus will be $0.00.

In short, the closer you are to the actual outcome, the more money you will earn.

Any bonus you earn will be paid on top of the $1.70. You'll only of course get this once the election results are out.

After indicating your prediction, you will be asked to express your *confidence* in it.
You have **2 options: Low** confidence and **High** confidence.

Your confidence influences the chance that a **part A prediction** will be the one that is chosen for bonus payment.

If you express **Low confidence** in your prediction, it will have a *normal chance* to be the one that is selected for the **bonus payment**.

If you express **High confidence** in your prediction, it will be *twice more likely* to be the one that is selected for the **bonus payment**.

We will select **only one** of your **part A** decisions for payment. Which one that is depends on your confidence ratings.

Once you express your confidence in your prediction, **part A** of the round is over, and **part B** begins.

In **part B** you will observe the **part A** prediction of another MTurker.

Over 200 MTurkers participated in a previous session in which they completed this task.

In each round, you can observe the part A prediction of **one** of these **previous MTurkers.**

Their prediction was made for the **same state you will see in that round.**
They could also earn a higher bonus if their estimate was more accurate.

Next to their prediction, you will also see whether they expressed **Low** or **High** **confidence in their prediction.**

Like you, if they expressed **High** **confidence** in a prediction, that prediction was *twice* more likely to be selected for payment.

Please note: each round you will see the prediction of a different MTurker.

In **part B** you have to make a second prediction.

You can make the *same* prediction as in part A, or *adjust* it as you wish.

Once you have made your **part B** prediction, the round is over and a new round begins.

IMPORTANT: You can win an additional bonus of $1.00 for your part B prediction.
This bonus will be calculated in the *same way* as your part A bonus:
one of your predictions will be randomly selected, and the closer the prediction is to the real outcome, the higher your bonus will be.

In total, you can win up to $2.00 in bonus ($1.00 for part A, and $1.00 for part B).

Click **Next** for a ***full*** practice round.
